# Supplementary material for: Single-crosslink microscopy in a biopolymer network dissects local elasticity from molecular fluctuations
Source: Nat Commun. 2019 Jul 25;10:3314. doi: 10.1038/s41467-019-11313-7 (PMC6658493; doi:10.1038/s41467-019-11313-7)
Supplement: Supplementary file 1 — Supplementary Information [file 41467_2019_11313_MOESM1_ESM.pdf]

Supplementary Information

Single-crosslink microscopy in a biopolymer network dissects local elasticity from molecular fluctuations

Lingxiang Jiang,* Qingqiao Xie, Boyce Tsang, and Steve Granick*

*Correspondence to: jianglx@jnu.edu.cn and sgranick@ibs.re.kr

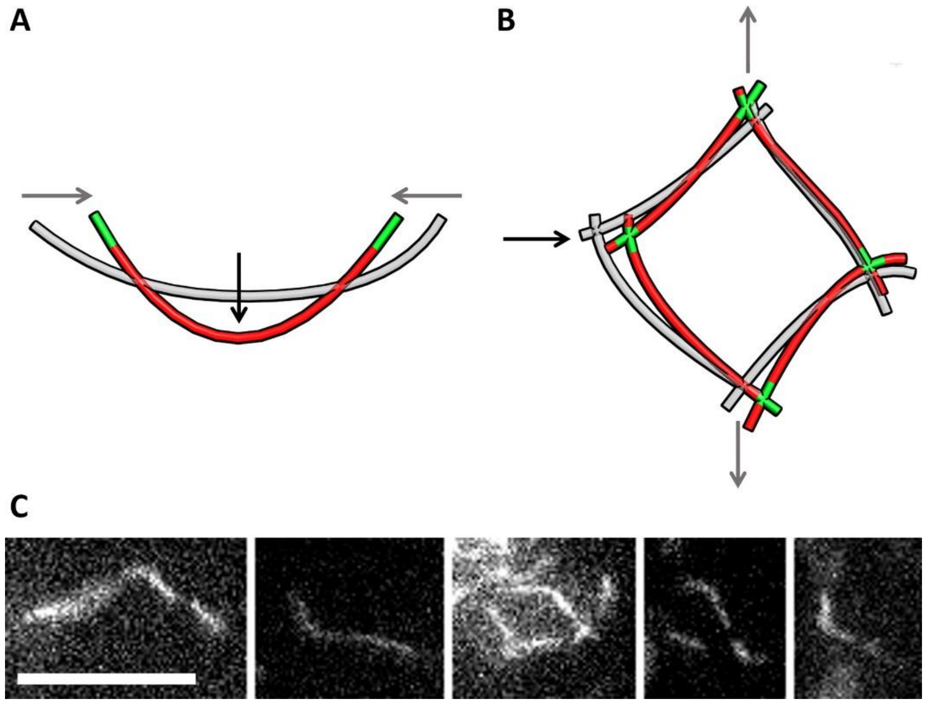

Supplementary Figure 1. Possible causes for anticorrelations.

A, Bending modes may cause two crosslinks move against (or away from) each other. **B**, In a rhombus configuration, motion of the left crosslink may cause the upper and lower crosslinks to move against (or away from) each other. **C,** Examples of actin filaments with unrelaxed or kinked configurations. Scale bar: 10 μm.

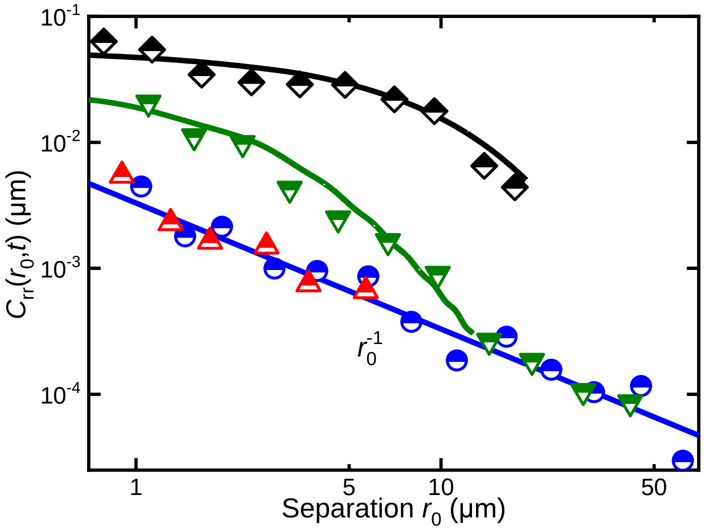

Supplementary Figure 2. Cross-correlation C_{rr} compared in crosslinked and uncrosslinked actin systems. For pairs of labeled actin segments, their separation r_0 and cross-correlation C_{rr} (defined in eq. 15) are recorded and binned per r_0 values. In an entangled, uncrosslinked network (actin concentration 0.5 mg ml^{-1}), intra-filament correlation (black points) is strong and drops little as a result of stiff single-filament mechanics; inter-filament correlation (green points) decays in a nonhydrodynamic way (deviating from $1/r_0$) due to the emergence of inter-filament structural forces up to the filament length (~ 15 µm) and subsequently transitions to hydrodynamic continuum behavior due to vanishing of the structural forces. For details, please refer to our earlier publication.[17] In the current case of crosslinked actin networks, the situation is drastically different, where inter and intra-filament correlations (blue and red points) collapse onto a single $1/r_0$ line over the entire measurable range, up to 50 µm.
